# Supplementary material for: Nuclear translocation of p85β promotes tumorigenesis of PIK3CA helical domain mutant cancer
Source: Nat Commun. 2022 Apr 13;13:1974. doi: 10.1038/s41467-022-29585-x (PMC9007954; doi:10.1038/s41467-022-29585-x)
Supplement: Supplementary file 1 — Supplementary materials [file 41467_2022_29585_MOESM1_ESM.pdf]

Supplementary Information

Nuclear translocation of p85β promotes tumorigenesis of *PIK3CA* helical domain mutant cancer

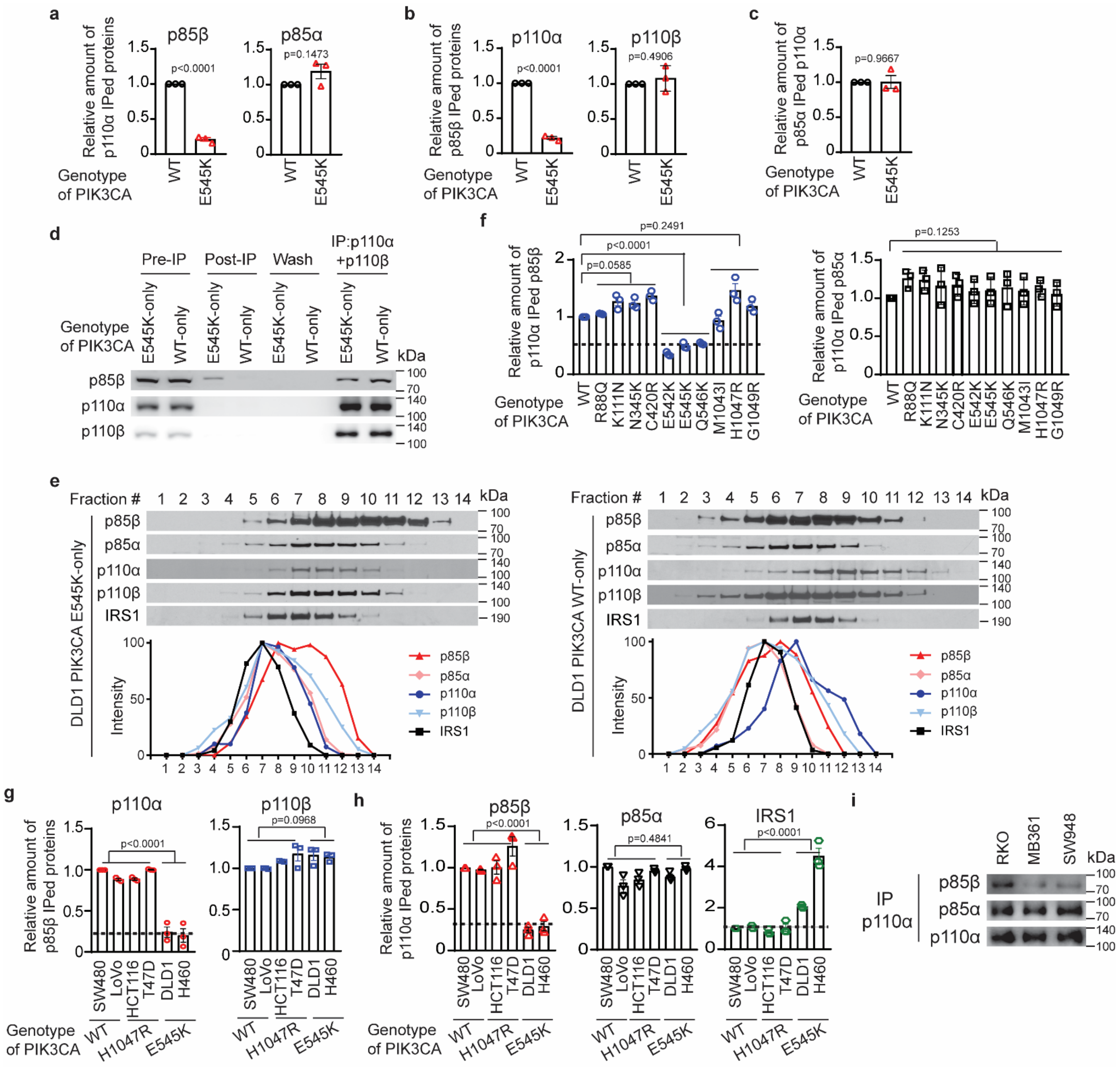

Figure S1. p85β disassociates from p110α helical domain mutated PI3K complex

(a) Quantification of Western blots shown in Fig 1b by Image J.

(b) Quantification of Western blot shown in Fig 1c by Image J.

(c) Quantification of Western blot shown in Fig 1d by Image J.

(d & d) *PIK3CA* helical domain mutant cells have more p110-free p85β. (d) Cell lysates were immunoprecipitated with both anti-p110α and anti-p110β antibodies and blotted with indicated antibodies. Pre-IP indicates cell lysates before immunoprecipitation; Post-IP indicates cell lysates after immunoprecipitation; Wash indicates buffers after washing beads; IP: p110α+p110β indicates immune complexes on the beads after immunoprecipitation. (e) Indicated cell lysates were fractionated with a Sepharose 6B column. The indicated elution fractions were blotted with the indicated antibodies, and the intensities of each protein at the indicated fractions were quantified with the Image J software.

(f) Quantification of Western blot shown in Fig 1f by Image J.

(g) Quantification of Western blot shown in Fig 1g by Image J.

(h) Quantification of Western blot shown in Fig 1h by Image J.

(**i**) p85β disassociates from PI3K complexes in *PIK3CA* helical domain mutant cells. Cell lysates from indicated cell lines were immunoprecipitated with an anti-p110α antibody and blotted with indicated antibodies.
The student's *t*-test (two-tailed) was used for statistical analyses. Data are presented as mean ± SEM of three independent experiments. Source data are provided as a Source Data file.

a

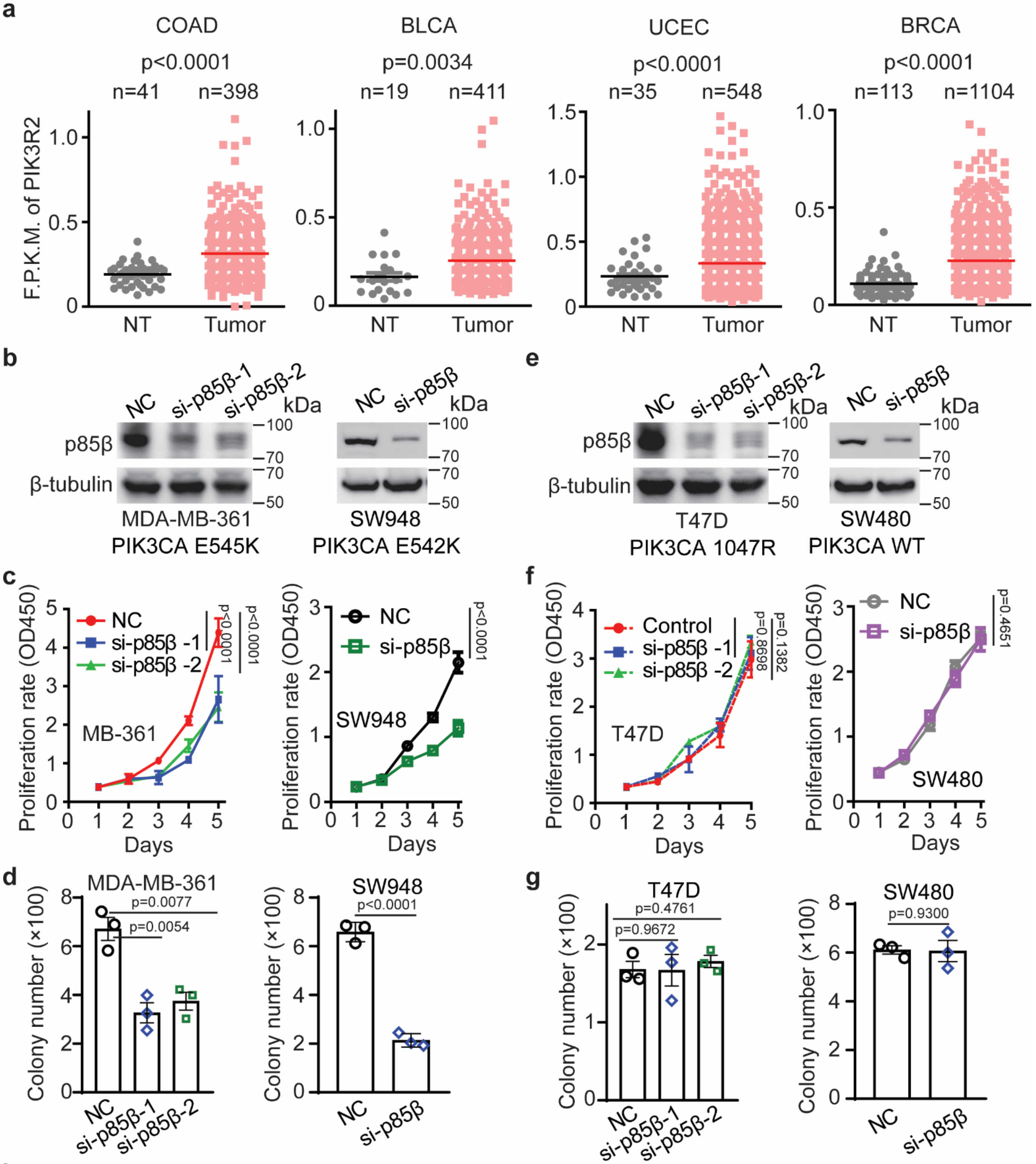

h

Cell lines / Gene mutations	DLD1 E545K-only	MB-361	H460	SW948	DLD1 WT-only	T47D	RKO	SW480
PIK3CA	E545K	E545K	E545K	E542K	WT	H1047R	H1047R	WT
PTEN	WT	NA	WT	NA	WT	WT	WT	WT
K-Ras	G13D	WT	Q61H	Q61L	G13D	WT	WT	G12V
BRAF	WT	WT	WT	NA	WT	WT	V600E	WT
TP53	WT	NA	WT	T118Qfs*5	WT	L194F	WT	R273H
CTNNB1	WT	NA	WT	NA	WT	WT	K312K	NA

Figure S2. Depletion of p85β specifically impairs the growth of cancer cells with a *PIK3CA* helical domain mutation.

(a) *PIK3R2* expression levels are higher in tumors compared to corresponding non-tumor tissue (NT). The RNA-seq data of tumors and matched non-tumor tissue were downloaded from the TCGA website. COAD: colon adenocarcinoma; BLCA: bladder carcinoma; UCEC: endometrial carcinoma; BRCA: breast cancer; FPKM: fragments per kilobase of exon per million reads mapped.

(b-g) Knockdown of p85β impair the growth of cancer cells a *PIK3CA* a helical domain mutation (E545K, or E542K), but not cells with a *PIK3CA* H1047R mutation or wild-type *PIK3CA*. p85β was knocked down with siRNA in the indicated cell lines, and the cells were assayed for: Western blot analyses of p85β protein (a, e); cell proliferation (c, f); colony formation (d, g).

(h) Mutation status of the indicated genes in cell lines used in this study. The data were obtained from COSMIC. NA: the indicated information is not available.

The student's *t*-test (two-tailed) was used for statistical analyses. Data are presented as mean ± SEM of three independent experiments. Source data are provided as a Source Data file.

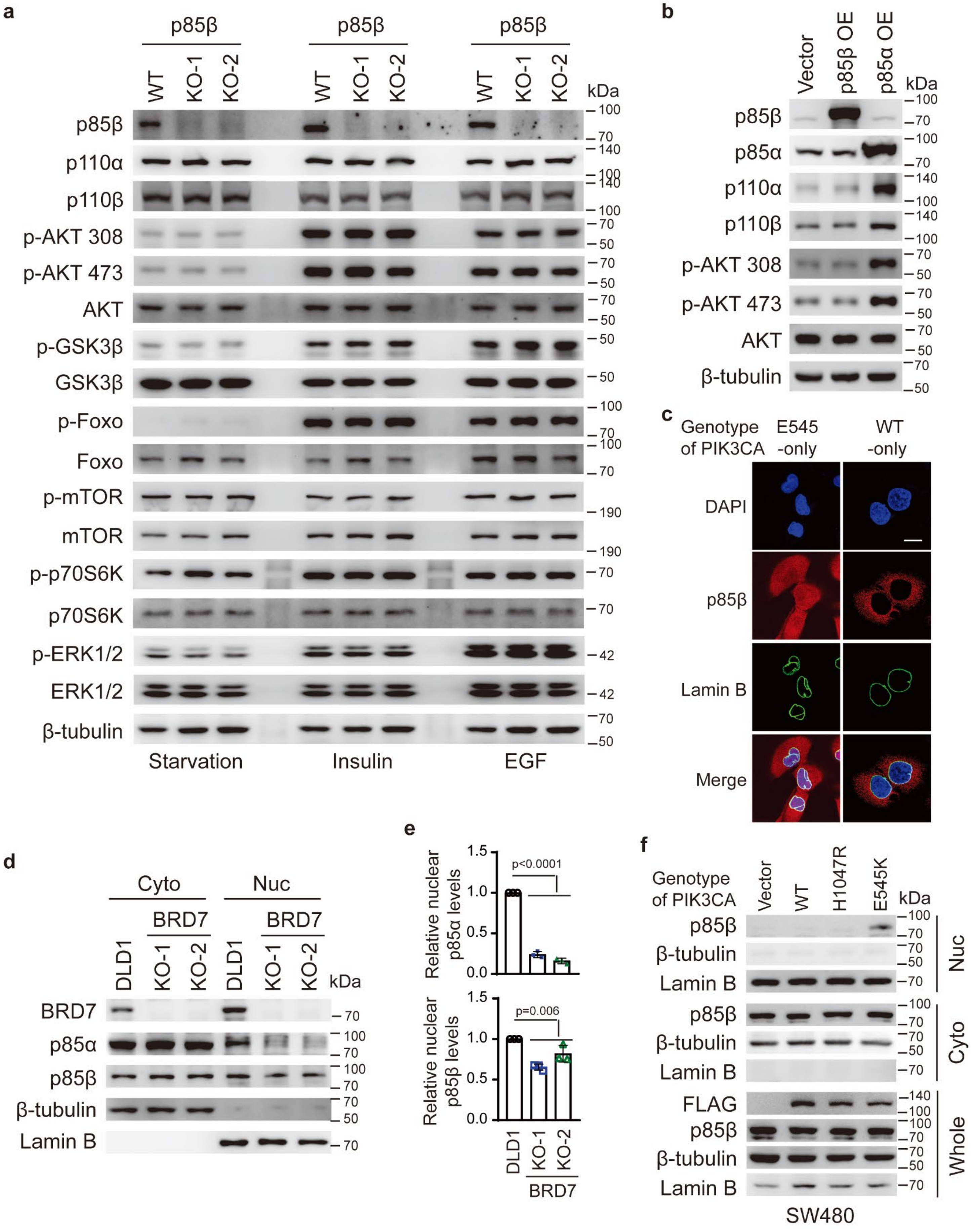

a
p85β
WT
KO-1
KO-2
kDa
p85β
p110α
p110β
p-AKT 308
p-AKT 473
AKT
p-GSK3β
GSK3β
p-Foxo
Foxo
p-mTOR
mTOR
p-p70S6K
p70S6K
p-ERK1/2
ERK1/2
β-tubulin
100
70
140
100
190
50
42
Starvation
Insulin
EGF
b
Vector
p85β OE
p85α OE
kDa
p85β
p85α
p110α
p110β
p-AKT 308
p-AKT 473
AKT
β-tubulin
c
Genotype of PIK3CA
E545 -only
WT -only
DAPI
p85β
Lamin B
Merge
d
Cyto
Nuc
BRD7
DLD1
KO-1
KO-2
kDa
BRD7
p85α
p85β
β-tubulin
Lamin B
e
Relative nuclear p85α levels
p<0.0001
Relative nuclear p85β levels
p=0.006
1.5
1.0
0.5
0.0
DLD1
KO-1
KO-2
BRD7
f
Genotype of PIK3CA
Vector
WT
H1047R
E545K
kDa
p85β
β-tubulin
Lamin B
FLAG
Nuc
Cyto
Whole
SW480

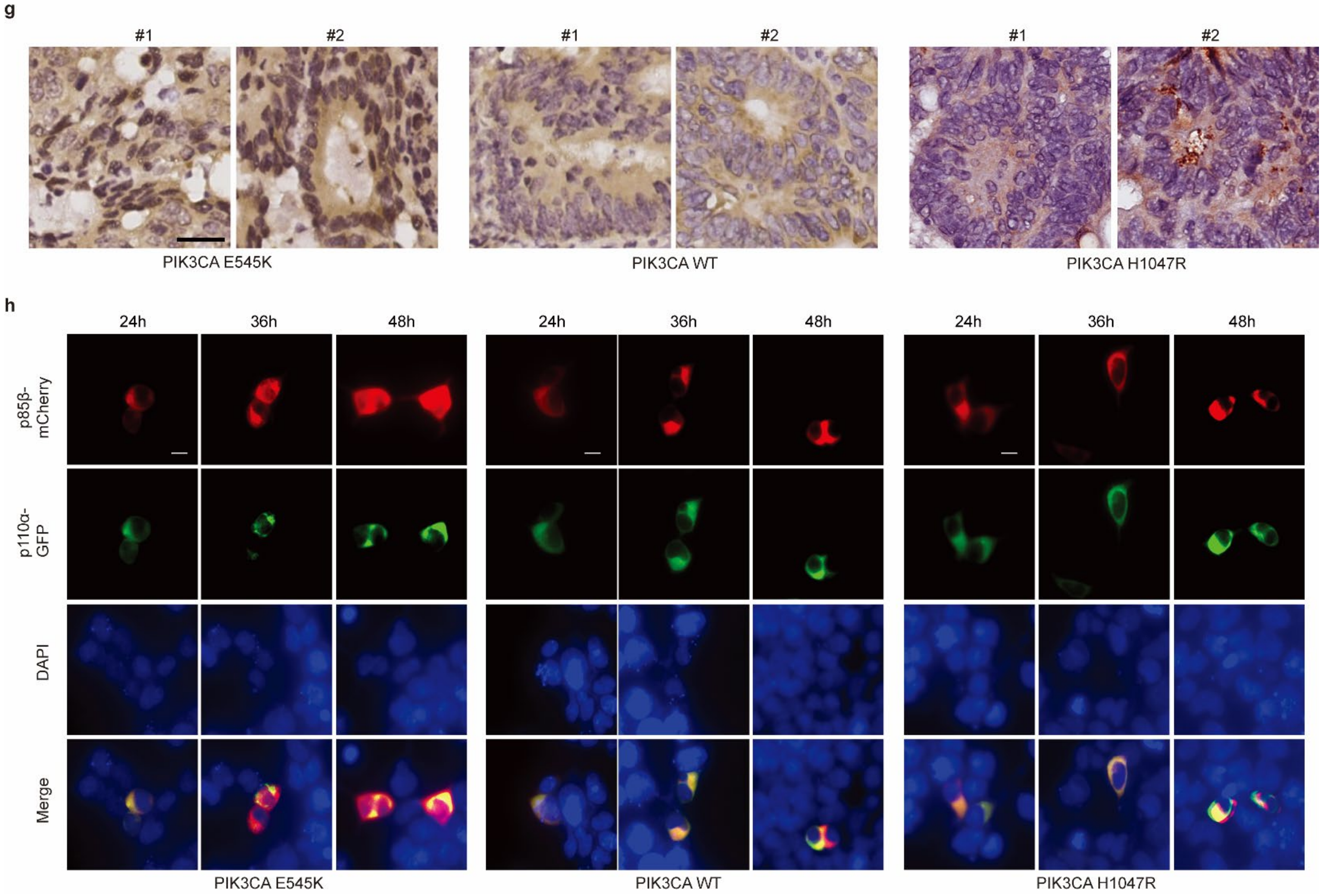

Figure S3. *PIK3CA* E545K mutation promotes the nuclear translocation of p85β.

(a & b) Knockout or overexpression of p85β have no impact on p110 stability and AKT signaling. The parental and p85β KO cells were grown in 6-well plates. After serum starvation (16 hours), cells were treated with insulin (1μg/ml for 15 minutes) or EGF (200 ng/ml for 15 minutes). Cell lysates were blotted with the indicated antibodies (a). Overexpression of p85α, but not p85β, stabilizes p110α protein and increases phosphorylation of AKT. DLD1 *PIK3CA* E545K-only cells were transfected with HA-tagged p85β or p85α. Three days after transfection, lysates from indicated cell lines were harvested and blotted with indicated antibodies (b).

(**c**) p85β is localized in the nucleus in *PIK3CA* E545K mutant cells, but not in *PIK3CA* WT cells. Cells of the indicated genotypes were stained with antibodies against p85β and lamin B and DAPI. Scale bar = 10μm.

(d & e) Knockout of BRD7 abolishes nuclear translocation of p85α but not p85β. BRD7 gene was knocked out in DLD cells using the CRISPR/Cas9 system. DLD1 cells and BRD7 KO cells were fractionated and blotted with indicated antibodies (d). Quantification of Western blots is shown in Figure S3C by Image J (e).

(f) SW480 was transfected with FLAG-tagged wild-type *PIK3CA*, *PIK3CA* H1047R, or E545K mutant construct. Three days post-transfection, cell lysates were fractionated and blotted with indicated antibodies.

(g) Immunohistochemistry images of p85β staining in colorectal tumors with *PIK3CA* E545K mutation, wild-type *PIK3CA*, or *PIK3CA* H1047R mutation. Scale bar = 20 μm.

(**h**) Ectopically expressed p85β is localized in the nucleus when it is co-expressed with *PIK3CA*/p110α E545K, but not co-expressed with WT *PIK3CA*/p110α or *PIK3CA*/p110α H1047R. A construct expressing p85β-mCherry fusion protein was co-expressed with p110α E545K-GFP, WT p110α-GFP, or p110α H1047R-GFP in SW480 cells, which harbor WT *PIK3CA*. Cells were imaged at the indicated time points. Scale bars = 10 μm.

The student's *t*-test (two-tailed) was used for statistical analyses. Data are presented as mean ± SEM of three independent experiments. Source data are provided as a Source Data file.

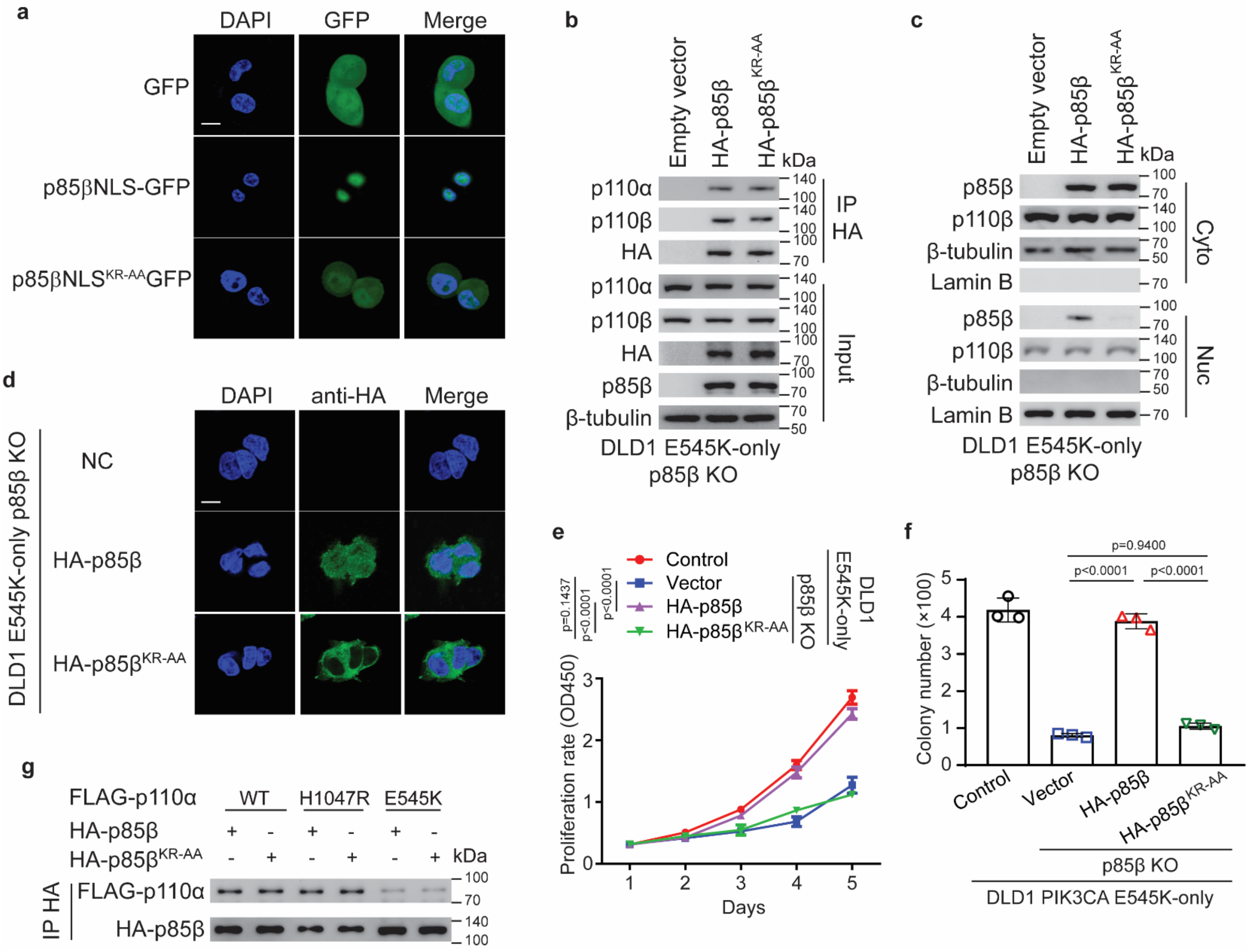

Figure S4. Nuclear localization signal (NLS) is critical for nuclear translocation of p85β.

(a) The p85β NLS drives nuclear localization of GFP. The WT p85β NLS or p85β NLS mutant were fused with GFP, expressed in SW480 cells, and visualized under a fluorescent microscope. Scale bar = 10μm.

(b) The p85β NLS mutant does not impact its interaction with p110α and p110β. The DLD1 *PIK3CA* E545K-only p85β KO cells were transfected with either HA-tagged p85β or HA-tagged p85β$^{KR\text{-}AA}$. Stable expression clones were selected. Cell lysates were immunoprecipitated with anti-HA agarose and blotted with the indicated antibodies.

(c & d) NLS of p85β is critical for its nuclear translocation. The indicated cells were fractionated and blotted with indicated antibodies (c). The p85β subcellular localization in the indicated cell lines was evaluated by immunofluorescent staining (d). Scale bar = 10μm.

(e & f) Reconstitution of wild-type p85β, but not the p85β$^{KR\text{-}AA}$ mutant, rescues growth defects caused by p85β depletion in DLD1 *PIK3CA* E545K cells. The growth curve (d) and colony formation (e) of indicated cell lines are shown.

(g) KR-AA mutation has no impact on p85β-p110α interaction: the indicated FLAG-tagged p110α and HA-tagged p85β constructions were co-transfected into 293T cells. Cell lysates were immunoprecipitated with anti-HA agarose and then blotted with indicated antibodies.

Statistical analyses, two-way ANOVA was used for e, and student's *t*-test (two-tailed) was used for f. Data are presented as mean ± SEM of three independent experiments. Source data are provided as a Source Data file.

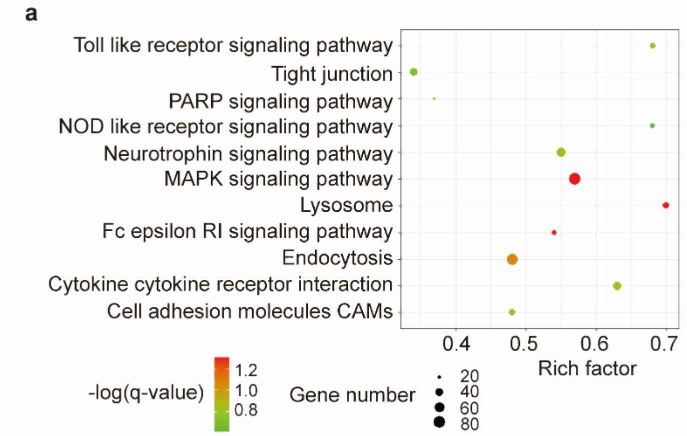

b

Genes	FC(KI/DLD1)	Regulation	Gene description	Pathways
ATM	13.34	up	ATM serine/threonine kinase	DNA damage
BRCA1	3.00	up	BRCA1, DNA repair associated	
BRCA2	7.19	up	BRCA2, DNA repair associated	
TGFBR3	8.46	up	transforming growth factor beta receptor 3	TGF beta signaling
TGFBI	3.46	up	transforming growth factor beta induced	
SMAD4	3.92	up	SMAD family member 4	
KDM6A	9.40	up	lysine demethylase 6A	Histone modification
KMT2C	20.01	up	lysine methyltransferase 2C	
KMT2D	3.11	up	lysine methyltransferase 2D	
SETD2	11.74	up	histone lysine methyltransferase	
SIRT1	3.96	up	sirtuin 1	
CDH1	3.39	up	cadherin 1, E-cadeherin	Wnt pathway
APC	14.04	up	adenomatous polyposis coli	
WNT10A	0.07	down	Wnt family member 10A	
WNT6	0.07	down	Wnt family member 6	

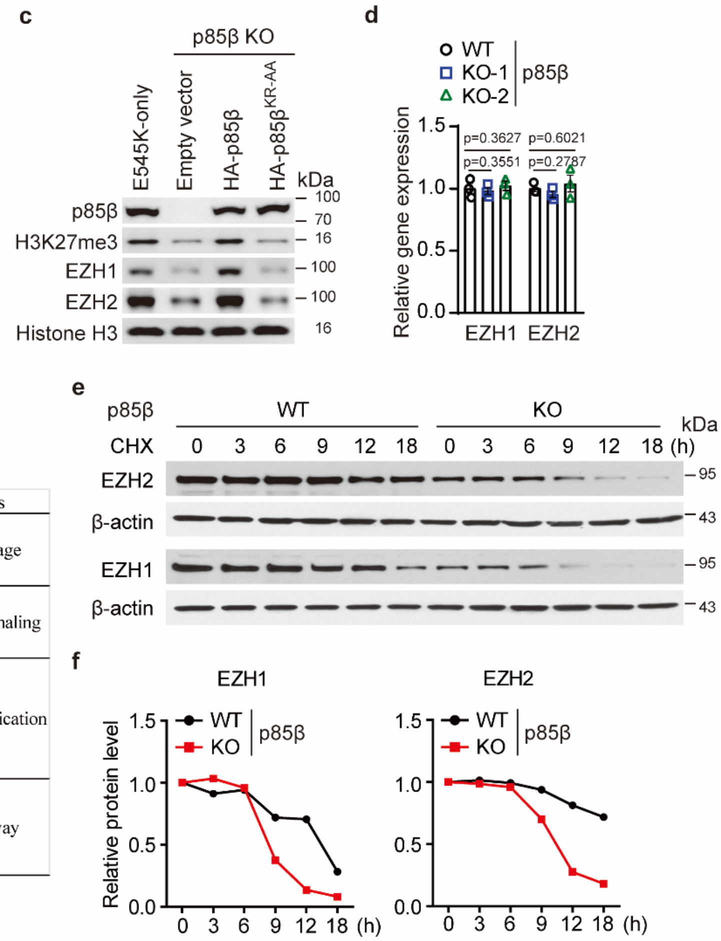

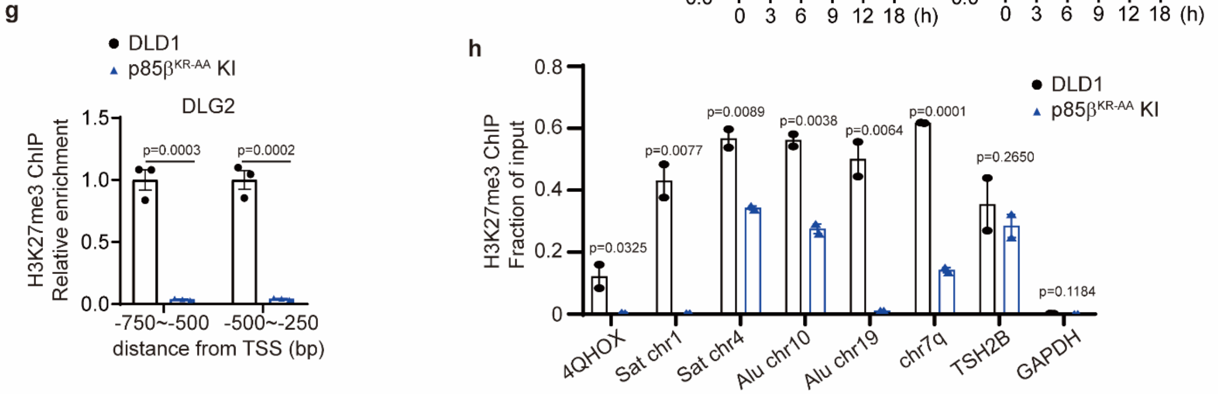

Figure S5. Nuclear p85β stabilizes EZH1/EZH2 to enhance H3K27me3 to regulate gene expression. (**a**) Pathway analysis of the RNA-seq data is shown in Figure 5a. (**b**) List of known tumor-suppressor genes that are up-regulated and known oncogenes that are down-regulated in the p85β NLS mutant cells. (**c**) Reconstitution of WT p85β, but not the NLS mutant, restores EZH1, EZH2, and H3K27me3 in p85β knockout cells. DLD1 *PIK3CA* E545K-only p85β knockout cells were transfected with the indicated constructs. Cell lysates were blotted with the indicated antibodies. (**d**) Knockout of p85β has no effect on mRNA levels of EZH1 and EZH2. Expression of EZH1 and EZH2 in DLD1 *PIK3CA* E545K-only and DLD1 *PIK3CA* E545K-only p85β KO cells were analyzed by quantitative RT-PCR. (**e & f**) EZH 1 and EZH2 proteins are less stable in p85β knockout E545K-only cells. DLD1 E545K-only and its p85β knockout derivative cells were treated with cycloheximide (CHX) for the indicated times. Cell lysates were blotted with the indicated antibodies. Western blots are shown in the upper panel (e), and quantification of EZH1 and EZH2 protein levels by imageJ are shown in the lower panel (f). Similar results have been repeated twice. (**g**) ChIP-PCR of H3K27me3 in the promoter region of the DLG2 gene in parental DLD1 and p85β NLS mutant KI cells. (**h**) ChIP-PCR of H3K27me3 in heterochromatin regions in parental DLD1 and p85β NLS mutant KI cells. 4QHOX: D4Z4 repeat sequences on chromosome 4; Sat chr1: satellite sequences on chromosome 1; Sat chr4: satellite sequences on chromosome 4; Alu chr10: Alu sequences on chromosome

10; Alu chr19: Alu sequences on chromosome 19; chr7q: the telomeric TTAGGC repeats at the chromosome 7q; TSH2B: the promoter region of testis-specific histone 2B variant; GAPDH: the promoter region of GAPDH (negative control). The student's *t*-test (two-tailed for d&g, one-tailed for h) was used for statistical analyses. Data are presented as mean ± SEM of three (d&g) or two (h) independent experiments. Source data are provided as a Source Data file.

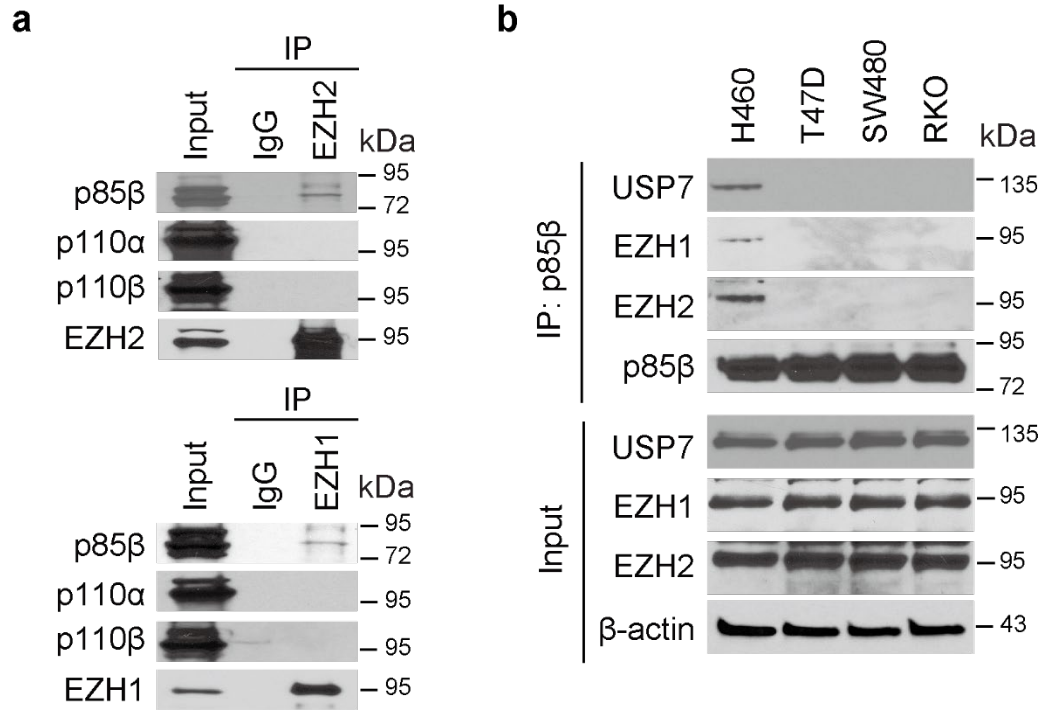

Figure S6. Nuclear p85β increases tri-methylation of H3K27 by stabilizing EZH1/2 proteins.

(a) Nuclear p85β interacts with USP7, EZH1, and EZH2. DLD1 cells were lysed and immunoprecipitated (IP) with either an anti-EZH1 or an anti-EZH2 antibody, then blotted with indicated antibodies.

(b) Nuclear p85β interacts with USP7, EZH1, and EZH2 only in cells with a *PIK3CA* helical domain mutation (H460), but not in cells with either WT *PIK3CA* (SW480), or a *PIK3CA* H1047R mutation (T47D and RKO).

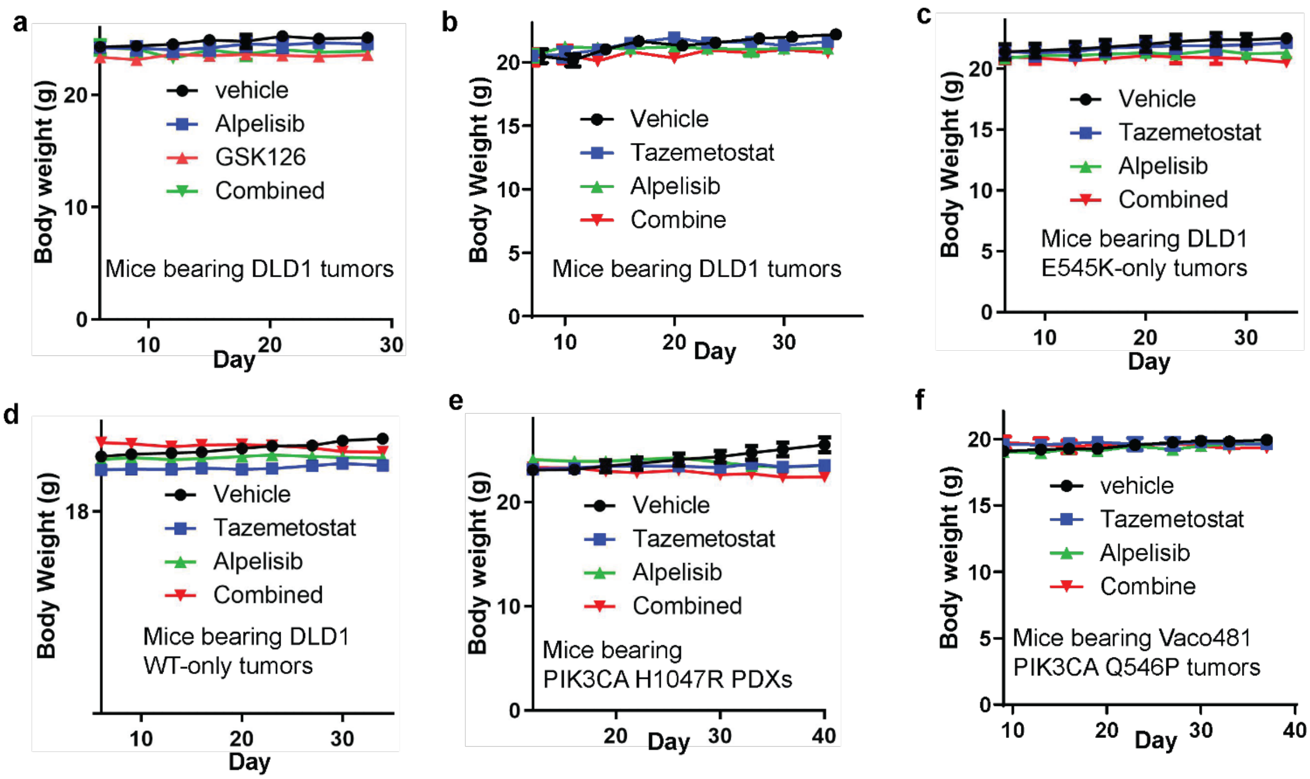

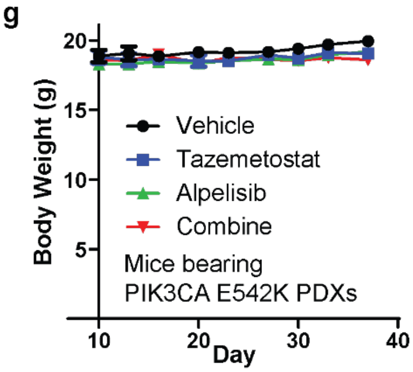

h

	fig7a	fig7b	fig7c	fig7d	fig7e	fig7f	fig7g
Combination ratio	9.84	6.26	4.71	0.99	1.07	6.64	8.98
Synergy	√	√	√			√	√
Additive effect				√	√		
Antagonism							

Figure S7. (**a** to **g**) Body weights of mice treated were maintained during the course of treatment shown in Figure 7**.** (**h**) Synergistic analyses of the drug combination on various CRC models shown in Figure 7. Source data are provided as a Source Data file.

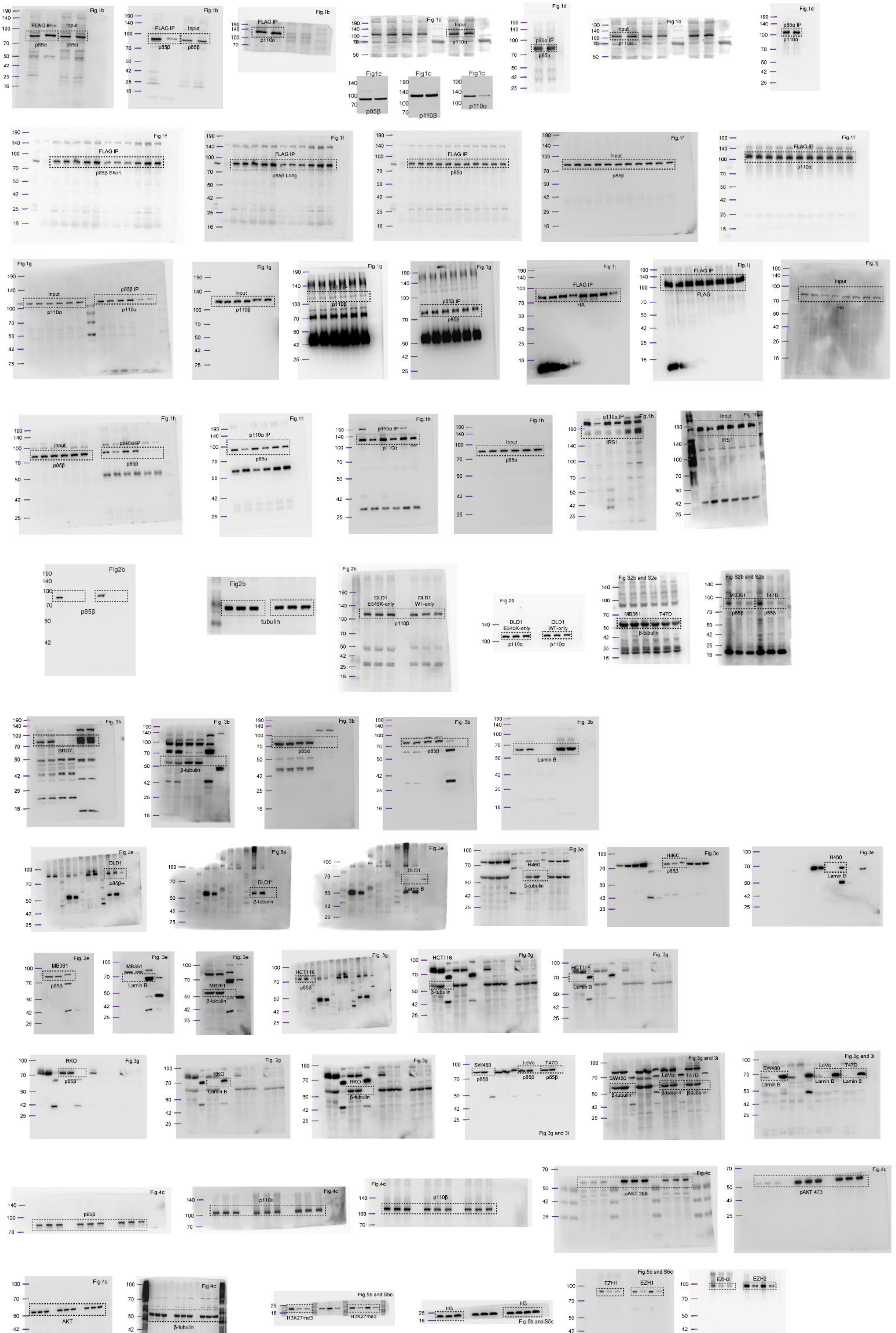

Fig.1b
FLAG IP
Input
p85α
p85β
p110α
Fig.1c
p110α
Fig1c
p85β
p110β
Fig.1d
p85α IP
Fig.1f
FLAG IP
p85β Short
p85β Long
Fig.1g
p85β IP
p110β
p85β
Fig.1j
HA
FLAG
Fig.1h
p110α IP
IRS1
Fig2b
tubulin
Fig.2b
DLD1
E545K-only
WT-only
Fig S2b and S2e
MB361
T47D
β-tubulin
Fig. 3b
BRD7
Lamin B
Fig.3e
H460
Fig. 3g
HCT116
RKO
SW480
LoVo
Fig.3g and 3i
Fig.4c
pAKT 308
pAKT 473
AKT
Fig.5b and S5c
H3K27me3
H3
EZH1
EZH2

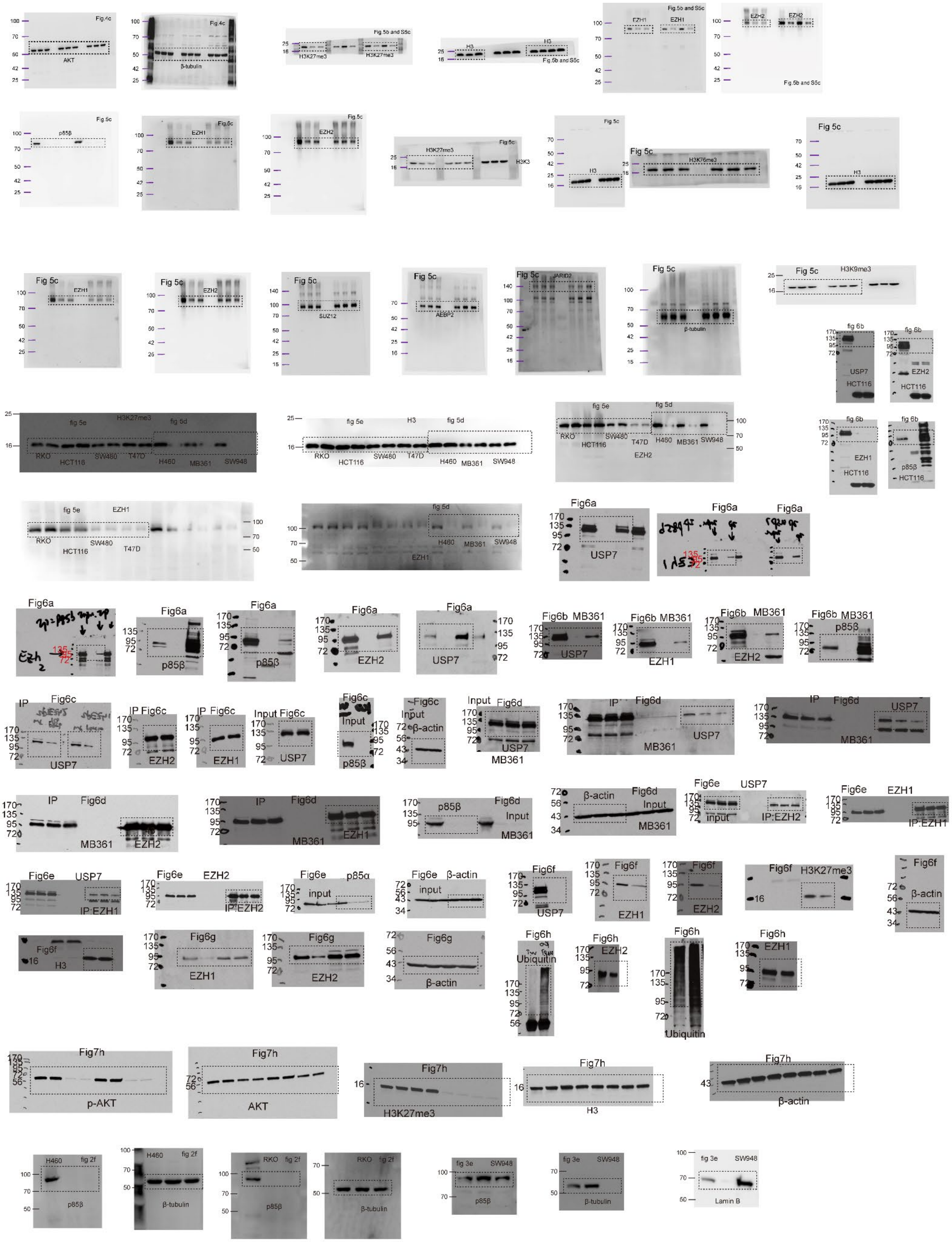

Fig.4c
AKT
β-tubulin
Fig.5b and S5c
H3K27me3
H3
EZH1
EZH2
Fig.5c
p85β
H3K76me3
Fig 5c
SUZ12
AEBP2
JARID2
H3K9me3
fig 6b
USP7
HCT116
fig 5e
fig 5d
RKO
SW480
T47D
H460
MB361
SW948
Fig6a
Fig6b MB361
Fig6c
IP
Input
Fig6d
Fig6e
IP:EZH2
IP:EZH1
input
p85α
β-actin
Fig6f
Fig6g
Fig6h
Ubiquitin
Fig7h
p-AKT
fig 2f
fig 3e
Lamin B

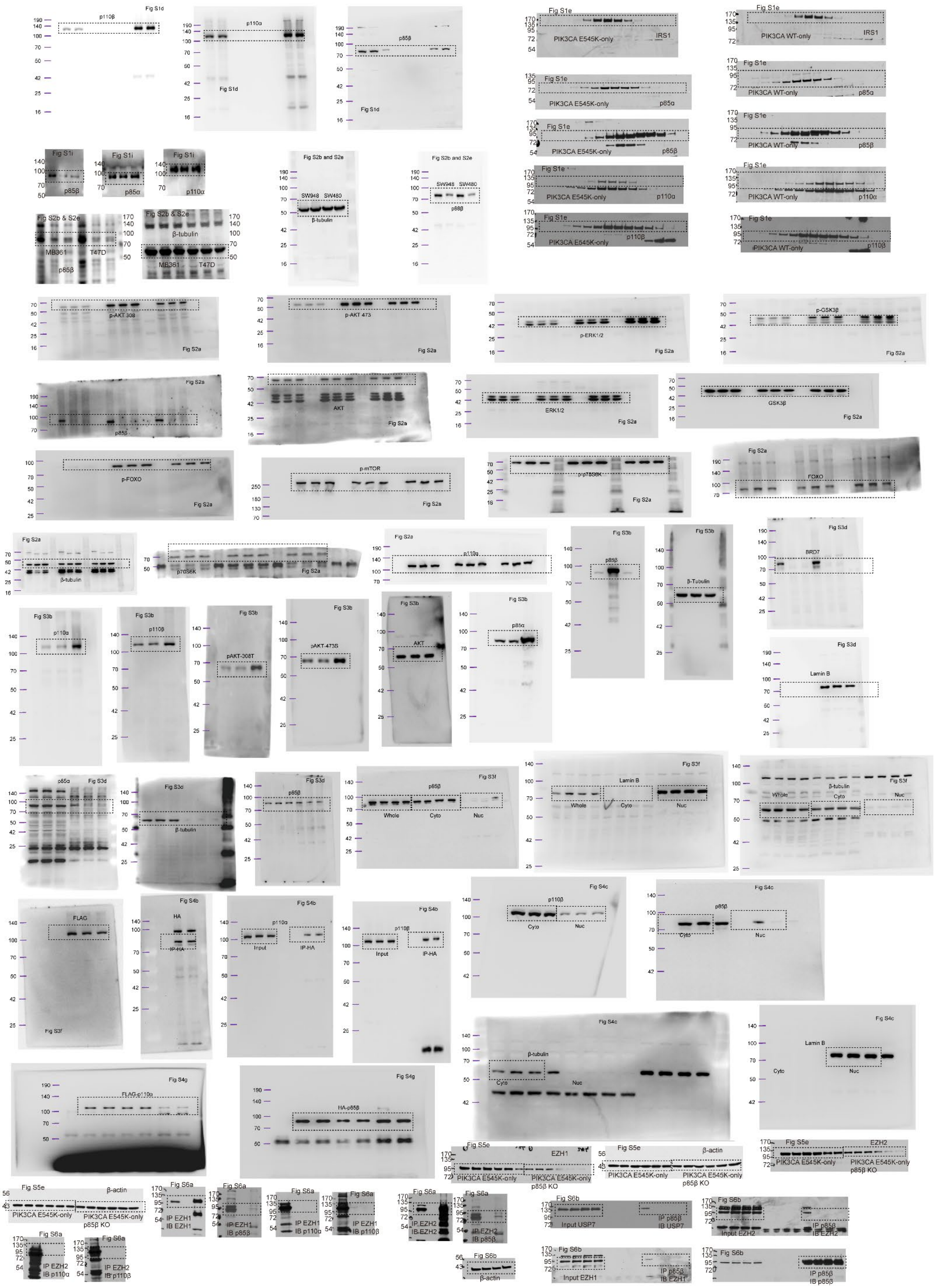

Fig S1d
p110β
p110α
p85β
Fig S1e
PIK3CA E545K-only
IRS1
PIK3CA WT-only
p85α
p85β
p110α
p110β
Fig S1i
Fig S2b and S2e
SW948
SW480
β-tubulin
Fig S2b & S2e
MB361
T47D
Fig S2a
p-AKT 308
p-AKT 473
p-ERK1/2
p-GSK3β
AKT
ERK1/2
GSK3β
p-FOXO
p-mTOR
FOXO
p70S6K
Fig S3b
pAKT-308T
pAKT-473S
β-Tubulin
Fig S3d
BRD7
Lamin B
Fig S3f
Whole
Cyto
Nuc
Fig S4b
FLAG
HA
IP-HA
Input
Fig S4c
Fig S4g
FLAG-p110α
HA-p85β
Fig S5e
EZH1
EZH2
β-actin
PIK3CA E545K-only p85β KO
Fig S6a
IP EZH1
IB EZH1
IB p85β
IB p110α
IB p110β
IP EZH2
IB EZH2
IP p85β
Fig S6b
Input USP7
IB USP7
Input EZH2
Input EZH1
IB EZH1
IB p85β

Figure S8. Original Western Blot images.

Table S1. Intersection of differentially expressed genes (DEGs) list from ChIP-seq and RNA-seq profiles

Gene symbol	Gene name
DLG2	discs large MAGUK scaffold protein 2
SLC6A15	solute carrier family 6 member 15
RAB3C	RAB3C, member RAS oncogene family
GFRA1	GDNF family receptor alpha 1
CPNE4	copine 4
NPTX1	neuronal pentraxin 1
FLT4	fms related tyrosine kinase 4
ZDBF2	zinc finger DBF-type containing 2
PCK1	phosphoenolpyruvate carboxykinase 1
SATB1	SATB homeobox 1
PRICKLE1	prickle planar cell polarity protein 1
SLC44A1	solute carrier family 44 member 1
JAM3	junctional adhesion molecule 3
LRRC58	leucine rich repeat containing 58
MAST4	microtubule associated Ser/Thr kinase family member 4
AUTS2	activator of transcription and developmental regulator
ISM2	isthmin 2
TAB2	TGF-beta activated kinase 1 (MAP3K7) binding protein 2
HELB	DNA helicase B
VCAN	versican
CLDN12	claudin 12
VSNL1	visinin like 1
TRIM36	tripartite motif containing 36
TCF4	transcription factor 4
SCN8A	sodium voltage-gated channel alpha subunit 8
PIP5K1B	phosphatidylinositol-4-phosphate 5-kinase type 1 beta
PTPN3	protein tyrosine phosphatase, non-receptor type 3
ENPP1	ectonucleotide pyrophosphatase/phosphodiesterase 1
TMTC1	transmembrane and tetratricopeptide repeat containing 1
RHOBTB1	Rho related BTB domain containing 1
SMARCA2	SWI/SNF related, matrix associated, member 2
PLEKHG1	pleckstrin homology and RhoGEF domain containing G1
RABGAP1L	RAB GTPase activating protein 1 like
PLAGL1	PLAG1 like zinc finger 1
NDRG4	NDRG family member 4
RUNX2	runt related transcription factor 2
MYO10	myosin X
LTBP1	latent transforming growth factor beta binding protein 1

Table S2. List of reagents

Antibodies and Reagents	Source	Identifier
**Antibodies**		
Mouse monoclonal antibody anti-FLAG	Sigma-Aldrich	Cat# F1804, RRID:AB_262044
Mouse monoclonal antibody anti-Myc	Santa Cruz	Cat# sc-40, RRID:AB_627268
Mouse polyclonal antibody anti-HA	Santa Cruz	Cat# sc-805, RRID:AB_631618
Rabbit monoclonal antibody anti-p110 alpha	Cell Signaling Technology	Cat# 4249, RRID:AB_2165248
Rabbit monoclonal antibody anti-p110 beta	Cell Signaling Technology	Cat# 3011, RRID:AB_2165246
Rabbit monoclonal antibody anti-p85 alpha	Abcam	Cat# ab191606
Rabbit monoclonal antibody anti-p85 alpha pY607	Abcam	Cat# ab182651, RRID:AB_2756407
Rabbit monoclonal antibody anti-p85 beta	Abcam	Cat# ab180967
Rabbit monoclonal antibody anti-p85 beta pY464	Abcam	Cat# ab138364
Rabbit polyclonal antibody anti-IRS1	Proteintech	Cat# 17509-1-AP, RRID:AB_10596914
Rabbit polyclonal antibody anti-BRD7	Proteintech	Cat# 51009-2-AP, RRID:AB_2259226
Rabbit polyclonal antibody anti-β-tubulin	Bioss	Cat# bs-4511R, RRID:AB_11114300
Rabbit monoclonal antibody anti-Lamin B	Bioss	Cat# bsm-33010M
Rabbit monoclonal antibody anti-AKT pT308	Cell Signaling Technology	Cat# 13038, RRID:AB_2629447
Rabbit monoclonal antibody anti-AKT pS473	Cell Signaling Technology	Cat# 4060, RRID:AB_2315049
Rabbit monoclonal antibody anti-AKT	Cell Signaling Technology	Cat# 9272, RRID:AB_329827
Rabbit polyclonal antibody anti-GSK-3β pS9	Cell Signaling Technology	Cat# 9336, RRID:AB_331405
Rabbit monoclonal antibody anti-GSK-3β	Cell Signaling Technology	Cat# 9315, RRID:AB_490890
Rabbit monoclonal antibody anti-FoxO1 pT24	Cell Signaling Technology	Cat# 9464, RRID:AB_329842
Mouse monoclonal antibody anti-FoxO1	Millipore	Cat# 3012276
Rabbit monoclonal antibody anti-mTOR pS2448	Cell Signaling Technology	Cat# 5536, RRID:AB_10691552
Rabbit monoclonal antibody anti-mTOR	Cell Signaling Technology	Cat# 2983, RRID:AB_2105622
Rabbit monoclonal antibody anti-p70 S6 kinase pS371	Cell Signaling Technology	Cat# 9208, RRID:AB_330990
Rabbit monoclonal antibody anti-anti-p70 S6 kinase	Cell Signaling Technology	Cat# 2708, RRID:AB_390722
Rabbit monoclonal antibody anti-Erk1/2 pT202, pT204	Cell Signaling Technology	Cat# 4370, RRID:AB_2315112
Rabbit monoclonal antibody anti-Erk1/2	Cell Signaling Technology	Cat# 4695, RRID:AB_390779
Rabbit monoclonal antibody anti-H3K4me3	Cell Signaling Technology	Cat# 9783, Tri-Methyl Histone H3 Antibody Sampler Kit
Rabbit monoclonal antibody anti-H3K9me3	Cell Signaling Technology	Cat# 9783, Tri-Methyl Histone H3 Antibody Sampler Kit
Rabbit monoclonal antibody anti-H3K27me3	Cell Signaling Technology	Cat# 9783, Tri-Methyl Histone H3 Antibody Sampler Kit
Rabbit monoclonal antibody anti-H3K36me3	Cell Signaling Technology	Cat# 9783, Tri-Methyl Histone H3 Antibody Sampler Kit
Rabbit monoclonal antibody anti-H3K79me3	Cell Signaling Technology	Cat# 9783, Tri-Methyl Histone H3 Antibody Sampler Kit
Rabbit monoclonal antibody anti-H3	Cell Signaling Technology	Cat# 9783, Tri-Methyl Histone H3 Antibody Sampler Kit
Rabbit monoclonal antibody anti-EZH1	Cell Signaling Technology	Cat# 62083, PRC2 Antibody Sampler Kit
Rabbit monoclonal antibody anti-EZH2	Cell Signaling Technology	Cat# 62083, PRC2 Antibody Sampler Kit
Rabbit monoclonal antibody anti-SUZ12	Cell Signaling Technology	Cat# 62083, PRC2 Antibody Sampler Kit
Rabbit monoclonal antibody anti-EED	Cell Signaling Technology	Cat# 62083, PRC2 Antibody Sampler Kit
Rabbit monoclonal antibody anti-JARID2	Cell Signaling Technology	Cat# 62083, PRC2 Antibody Sampler Kit
Rabbit monoclonal antibody anti-AEBP2	Cell Signaling Technology	Cat# 62083, PRC2 Antibody Sampler Kit
Rabbit polyclonal antibody anti-IgG	Cell Signaling Technology	Cat# 2729, RRID:AB_1031062
**Chemicals and reagents**		
Anti-Flag Affinity Gel	Bimake	Cat# B23101

Anti-Myc tag Mouse mAb conjugated Agarose Beads	Engibody Biotechnology	Cat# AT0080
Anti-HA tag Mouse mAb conjugated Agarose Beads	Engibody Biotechnology	Cat# AT0079
EGF	Sigma-Aldrich	Cat# E5036
Insulin	Sigma-Aldrich	Cat# I2643
Alpelisib	Selleck Chemicals	Cat# S2814
GSK126	Selleck Chemicals	Cat# S7061
Tazemetostat	Selleck Chemicals	Cat# S7128
MG132	Selleck Chemicals	Cat# S2619
DAPI	Sigma-Aldrich	Cat# D9542
**Critical Commercial Assay Kits**		
USER cloning system	NEB	Cat# #M5505L
Site-Directed Mutagenesis Kit	Agilent	Cat# 200523
EnVision-HRP kit	Dako	Cat# K4001
PrimeScript RT Reagent Kit	TAKARA	Cat# RR037A
Mycoplasma Detection Kit	Yeasen	Cat# 40601ES20

Table S3. List of primers.

Primer name	Primer sequences（5' to 3'）	
**Primers for qRT-PCR and ChIP-PCR**		
PIK3R2	Forward	AAAGGCGGGAACAATAAGCTG
	Reverse	CAACGGAGCAGAAGGTGAGTG
EZH1	Forward	ATGCGACTTCGACAACTTAAACG
	Reverse	GGCTTCATTGACTGAACAGGTT
EZH2	Forward	AATCAGAGTACATGCGACTGAGA
	Reverse	GCTGTATCCTTCGCTGTTTCC
DLG2 -1000	Forward	GGTGACTAGTTGAGTGGCCT
	Reverse	GGGAAAACAGACACCCAGGATT
DLG2 -750	Forward	GTGGAAGGTGGAGGATTTCAA
	Reverse	ATCTGCTGTCTTGGCAGACG
DLG2-500	Forward	GGTGTCAGGGAGAGGGAAAAG
	Reverse	CTCATTTGCTGAATGAGCGGT
4qHox	Forward	CGAGGACGGCGACGGAGAC
	Reverse	ACCCTGTCCCGGGTGCCTG
Satellite chr1	Forward	CATCGAATGGAAATGAAAGGAGTC
	Reverse	ACCATTGGATGATTGCAGTCAA
Satellite chr4	Forward	CTGCACTACCTGAAGAGGAC
	Reverse	GATGGTTCAACACTCTTACA
Alu chr10	Forward	GATTCTCAACAGCAGAATTCCATGCC
	Reverse	CATGTTTGAGAATGTCTACTTCTTAG
Alu chr19	Forward	CCACGTGTTTATCTGTAAGGTG
	Reverse	GTTAGGAGCTAGAAGGAGCCTG
Chr7q	Forward	CCTCGCTTTGACACGACTCGG
	Reverse	GCACAGGATTCAGACGGGCTTT
TSH2B	Forward	GCAGCACTGCCTGAATGTTA
	Reverse	TGTATTTGGCGGCAGTGTTA
GAPDH	Forward	TCTGCCCTCCTACCAGAAGA
	Reverse	TATTGAGGGCAGGGTGAGTC
β-tubulin	Forward	GGCCAAGGGTCACTACACG
	Reverse	GCAGTCGCAGTTTTCACACTC
**Primers for sgRNAs and siRNAs**		
PIK3R2	Forward	CACCGCCGGCCTCAACGAGCGCACA
sgRNA-1	Reverse	AAACTGTGCGCTCGTTGAGGCCGGC
PIK3R2	Forward	CACCGAGAACTGCTCGGGCAAGTCG
sgRNA-2	Reverse	AAACCGACTTGCCCGAGCAGTTCTC
PIK3R2	Forward	CACCGCCCACTGATCCACGTCGCTC
sgRNA-3	Reverse	AAACGAGCGACGTGGATCAGTGGGC
BRD7	Forward	CACCGTCGGACAAACACCTCTACG
sgRNA-1	Reverse	AAACCGTAGAGGTGTTTGTCCGAC
BRD7	Forward	CACCGAAGTCACCGAACTCTCCAC
sgRNA-3	Reverse	AAACGTGGAGAGTTCGGTGACTTC
BRD7	Forward	CACCGGAGGCAAGTCTAATCTCAC
sgRNA-3	Reverse	AAACGTGAGATTAGACTTGCCTCC
p85β	Forward	GGCUGGACAGCGAAUCUCAdTdT
siRNA-1	Reverse	UGAGAUUCGCUGUCCAGCCdTdT
p85β	Forward	GGAACAAUAAGCUGAUCAAdTdT
siRNA-2	Reverse	UUGAUCAGCUUAUUGUUCCdTdT
**Subcloning primers**		
FLAG-p110α	Forward	GGTCCCA/ideoxyU/TGCCTCCACGACCATCATCAG
WT	Reverse	GGCATAG/ideoxyU/TCAGTTCAATGCATGCTGTT
FLAG-p110α	Forward	GATGAAACAAGACAACTTTGTGACCTTCGG
R88Q	Reverse	CCGAAGGTCACAAAGTTGTCTTGTTTCATC
FLAG-p110α	Forward	CAACCGTGAAGAAAACATCCTCAATCGAGA
K111N	Reverse	TCTCGATTGAGGATGTTTTCTTCACGGTTG

FLAG-p110α N345K	Forward	GCAACCTACGTGAAAGTAAATATTCGAGAC
	Reverse	GTCTCGAATATTTACTTTCACGTAGGTTGC
FLAG-p110α C420R	Forward	GCTAAAGAGGAACACCGTCCATTGGCATGG
	Reverse	CCATGCCAATGGACGGTGTTCCTCTTTAGC
FLAG-p110α E542K	Forward	CGAGATCCTCTCTCTAAAATCACTGAGCAG
	Reverse	CTGCTCAGTGATTTTAGAGAGAGGATCTCG
FLAG-p110α E545K	Forward	CTCTCTGAAATCACTGCGCAGGAGAAAGAT
	Reverse	ATCTTTCTCCTGCGCAGTGATTTCAGAGAG
FLAG-p110α Q546K	Forward	CTGAAATCACTGAGAAGGAGAAAGATTTTC
	Reverse	GAAAATCTTTCTCCTTCTCAGTGATTTCAG
FLAG-p110α M1043I	Forward	CATGAAACAAATTAATGATGCACATCATGG
	Reverse	CCATGATGTGCATCATTAATTTGTTTCATG
FLAG-p110α H1047L	Forward	CAAATGAATGATGCACTTCATGGTGGCTGG
	Reverse	CCAGCCACCATGAAGTGCATCATTCATTTG
FLAG-p110α G1049R	Forward	GAATGATGCACATCATCGTGGCTGGACAAC
	Reverse	GTTGTCCAGCCACGATGATGTGCATCATTC
HA-p85α	Forward	CCGGAATTCATGAGTGCTGAGGGGTACCAG
	Reverse	ACGCGTCGACTCATCGCCTCTGCTGTGCATA
HA-p85β	Forward	CCGGAATTCATGGCGGGCCCTGAGGGCTTC
	Reverse	CCGCTCGAGTCAGCGGGCGGCAGGCGGCG
p85β NLS	Forward	CGCGGATCCCAGGACAAGAGCCGCGAGTATG
	Reverse	CCGGAATTCCTGCTCTTCAAAGATCTTGATAG
mCherry	Forward	CCGCTCGAGATGGTGAGCAAGGGCGAGGAGGA
	Reverse	CCGGGGCCCCTTGTACAGCTCGTCCATGCCGC
GFP	Forward	CGCGGATCCATGGTGAGCAAGGGCGAGGAG
	Reverse	CGCGGATCCCTTGTACAGCTCGTCCATGCC
HA-p85β KR477,478AA	Forward	CTCCCAGGAGCTGCAGATGGCGGCTACTGCAATTG AGGCCTTC
	Reverse	GAAGGCCTCAATTGCAGTAGCCGCCATCTGCAGCTC CTGGGAG
p85β KR477, 478AA Knockin vector	Left Arm forward	GGGAAAG/ideoxyU/CACAATGGCTCAAGCCTGTA
	Left Arm reverse	GGAGACA/ideoxyU/CCTGGGACTCCCCAAAAGGC
	Right Arm forward	GGTCCCA/ideoxyU/AGGTGCTGAGCTGCGCC
	Right Arm reverse	GGCATAG/ideoxyU/CTCTCATGGATCTCGGCAAT
	KR477, 478AA of p85b for genomic DNA and PAM mutation for gRNA, forward	GCGCCCACTCCTCCAGGAGCTGCAGATGGCGGCTA CTGCAATT
	KR477, 478AA of p85b for genomic DNA and PAM mutation for gRNA, reverse	AATTGCAGTAGCCGCCATCTGCAGCTCCTGGAGGA GTGGGCGC
	P1 for AAV and p85b screening, forward	TTTTGTCACTCAAGGACTGTGC
	P2 for AAV and p85b screening, reverse	TATGGAGCCGCCACTTACAC
	gRNA for p85b genomic DNA, forward	CACCGTCATCTGCAGCTCCTGGAGG
	gRNA for p85b genomic DNA, reverse	AAACCCTCCAGGAGCTGCAGATGAC
