# Supplementary material for: Nuclear translocation of p85β promotes tumorigenesis of PIK3CA helical domain mutant cancer
Source: Nat Commun. 2022 Apr 13;13:1974. doi: 10.1038/s41467-022-29585-x (PMC9007954; doi:10.1038/s41467-022-29585-x)
Supplement: Supplementary file 6 — Reporting Summary [file 41467_2022_29585_MOESM6_ESM.pdf]

Corresponding author(s): Yujun Hao and Zhenghe Wang

Last updated by author(s): 2022-3/1

Reporting Summary

Nature Portfolio wishes to improve the reproducibility of the work that we publish. This form provides structure for consistency and transparency in reporting. For further information on Nature Portfolio policies, see our Editorial Policies and the Editorial Policy Checklist.

Statistics

For all statistical analyses, confirm that the following items are present in the figure legend, table legend, main text, or Methods section.

n/a	Confirmed	
☐	☒	The exact sample size (*n*) for each experimental group/condition, given as a discrete number and unit of measurement
☐	☒	A statement on whether measurements were taken from distinct samples or whether the same sample was measured repeatedly
☐	☒	The statistical test(s) used AND whether they are one- or two-sided<br>*Only common tests should be described solely by name; describe more complex techniques in the Methods section.*
☒	☐	A description of all covariates tested
☒	☐	A description of any assumptions or corrections, such as tests of normality and adjustment for multiple comparisons
☐	☒	A full description of the statistical parameters including central tendency (e.g. means) or other basic estimates (e.g. regression coefficient) AND variation (e.g. standard deviation) or associated estimates of uncertainty (e.g. confidence intervals)
☐	☒	For null hypothesis testing, the test statistic (e.g. F, t, r) with confidence intervals, effect sizes, degrees of freedom and P value noted<br>*Give P values as exact values whenever suitable.*
☒	☐	For Bayesian analysis, information on the choice of priors and Markov chain Monte Carlo settings
☒	☐	For hierarchical and complex designs, identification of the appropriate level for tests and full reporting of outcomes
☒	☐	Estimates of effect sizes (e.g. Cohen's d, Pearson's r), indicating how they were calculated

Our web collection on statistics for biologists contains articles on many of the points above.

Software and code

Policy information about availability of computer code

Data collection	No software was used for data collection.
Data analysis	Image J, Prism graph pad, MACS2 (v1.4.2), bowtie (v1.1.0)

For manuscripts utilizing custom algorithms or software that are central to the research but not yet described in published literature, software must be made available to editors and reviewers. We strongly encourage code deposition in a community repository (e.g. GitHub). See the Nature Portfolio guidelines for submitting code & software for further information.

Data

Policy information about availability of data

All manuscripts must include a data availability statement. This statement should provide the following information, where applicable:

- Accession codes, unique identifiers, or web links for publicly available datasets
- A description of any restrictions on data availability
- For clinical datasets or third party data, please ensure that the statement adheres to our policy

Source data are provided with this paper as an excel file and original Western Blot images are provided in Figure S8. The RNA-seq and ChIP-seq data are deposited in the NCBI GEO database and the accession numbers are GSE190740 and GSE190434. Human reference genome hg19 was used as references for ChIP-seq data analyses. TCGA dataset were downloaded from http://xena.ucsc.edu/.

Field-specific reporting

Please select the one below that is the best fit for your research. If you are not sure, read the appropriate sections before making your selection.

☒ Life sciences ☐ Behavioural & social sciences ☐ Ecological, evolutionary & environmental sciences

For a reference copy of the document with all sections, see nature.com/documents/nr-reporting-summary-flat.pdf

Life sciences study design

All studies must disclose on these points even when the disclosure is negative.

Sample size	No statical method was used to predetermine sample size. Sample size and statistical analysis are provided in Figure legend.
Data exclusions	No data were excluded for the analyses.
Replication	At least triplicates were preformed for each data points. Details are provided in the source data. Asides from technical or reagent failures, the replication experiments were successful.
Randomization	For drug treatment experiments, mice are are randomly assigned to the treatment groups.
Blinding	The investigators were not blinded to allocation during the experiments and outcome assessment.

Reporting for specific materials, systems and methods

We require information from authors about some types of materials, experimental systems and methods used in many studies. Here, indicate whether each material, system or method listed is relevant to your study. If you are not sure if a list item applies to your research, read the appropriate section before selecting a response.

Materials & experimental systems

n/a	Involved in the study	
☐	☒	Antibodies
☐	☒	Eukaryotic cell lines
☒	☐	Palaeontology and archaeology
☐	☒	Animals and other organisms
☒	☐	Human research participants
☒	☐	Clinical data
☒	☐	Dual use research of concern

Methods

n/a	Involved in the study	
☐	☒	ChIP-seq
☒	☐	Flow cytometry
☒	☐	MRI-based neuroimaging

Antibodies

Antibodies used	Listed in table S2
Validation	The p85beta and USP7 antibodies were validated by showing that the knockout cells abrogated the proteins reacting with the antibodies. Other antibodies are either validated by the vendors or other investigators.

Eukaryotic cell lines

Policy information about cell lines

Cell line source(s)	The cell lines HEK 293, HCT116, DLD1, MB-361, H460, SW948, T47D, RKO and SW480were purchased from the ATCC.
Authentication	The cell lines were authenticated by the Genetica DNA Laboratories using STR profiling.
Mycoplasma contamination	All cell lines were tested routinely to avoid Mycoplasma contamination. The cell lines used in this study were not contaminated.
Commonly misidentified lines (See ICLAC register)	No commonly misidentified cell lines was used.

Animals and other organisms

Policy information about studies involving animals; ARRIVE guidelines recommended for reporting animal research

Laboratory animals	6 to 8-week old female Nu/J (Foxn1nu) mice from JAX (Strain #:002019) were used for the study. The housing light cycle is 12 light/12 dark. The room temp range is usually 68-79F for mice and humidity is usually 30-70%
Wild animals	No wild animals was used in the study
Field-collected samples	No field-collected samples was used in the study
Ethics oversight	All animal experiments were performed in accordance with protocols approved by the IACUC committee at Case Western Reserve University.

Note that full information on the approval of the study protocol must also be provided in the manuscript.

ChIP-seq

Data deposition

☒ Confirm that both raw and final processed data have been deposited in a public database such as GEO.

☒ Confirm that you have deposited or provided access to graph files (e.g. BED files) for the called peaks.

Data access links<br>*May remain private before publication.*	https://www.ncbi.nlm.nih.gov/geo/query/acc.cgi?acc=GSE190434
Files in database submission	DLD1_H3K27me3_IP.read1.fastq<br>DLD1_H3K27me3_IP.read2.fastq<br>DLD1 p85β K477A/R478A_H3K27me3_IP.read1.fastq<br>DLD1 p85β K477A/R478A_H3K27me3_IP.read2.fastq<br>DLD1_H3K27me3_IP_peaks.bw<br>DLD1 p85β K477A/R478A_H3K27me3_IP_peaks.bw
Genome browser session<br>(e.g. UCSC)	No longer applicable

Methodology

Replicates	H3K27me3 level in the cells was verified and DNA quality was confirmed by ChIP-qPCR with appropriate control before sending for ChIP-seq. No replicates used for ChIPs-seq analysis.
Sequencing depth	Sample TotalReadsCount UniquelyMappedReadsCount MappedRatio LengthOfReads Paired-OrSingle-End<br>DLD1_H3K27me3_ChIP 51079732 30247970 59.22% 33390472 Paired-end<br>DLD1 p85β K477A R478A_H3K27me3_ChIP 50260802 24875469 49.49% 123034552 Paired-end
Antibodies	H3K27me3, Cell Signaling Technology, 9733
Peak calling parameters	Unambiguously mapped reads were retained for subsequent generation of binding profiles, heatmaps and calling of peaks. MACS2 (v1.4.2) was used to identify regions in ChIPed samples of the signal enriched, and P < 1e-8 was used as the cutoff to identify statistically significant peaks. Mapped reads were visualized using the Integrative Genomics Viewer (IGV).
Data quality	FastQC (v0.11.2) was used for reads quality control.<br>P < 1e-8 was used as cutoff to identify peaks with MACS (v1.4.2).<br>Following reads quality control: Scan the read with a 4-base wide sliding window, cutting when the average quality per base drops below 15, drop reads which are less than 35% of initiation read length. Then reads quality was inspected using the FastQC software then output statistical result.<br>Sample PeakCount<br>DLD1_H3K27me3_ChIP 55340<br>DLD1 p85β K477A/R478A_H3K27me3_ChIP 22292
Software	Illumina Bcl2FastQ software used for basecalling. Raw reads were treated with Trimmomatic tools (v0.36) to remove adapters. The reads that passed the prefiltering step were aligned with the Bowtie2 (v2.4.2) software to the human genome (hg19). Peak calling was performed with MACS2 (v1.4.2). Homer software was used to annotate ChIP-seq peaks, and getDifferentialPeaks was used to find differential peaks between DLD1 parental cells and DLD1 p85β K477A/R478A mutant cells.
